# Long-term results of a study using individualized planning target volumes for hypofractionated intensity-modulated radiotherapy boost for prostate cancer

**DOI:** 10.1186/s13014-015-0400-1

**Published:** 2015-04-18

**Authors:** William Chu, D Andrew Loblaw, Kelvin Chan, Gerard Morton, Richard Choo, Ewa Szumacher, Cyril Danjoux, Jean-Philippe Pignol, Patrick Cheung

**Affiliations:** Department of Radiation Oncology, Odette Cancer Centre, Sunnybrook Health Sciences Centre, 2075 Bayview Ave., Toronto, ON M4N 3 M5 Canada; Department of Radiation Oncology, University of Toronto, Toronto, ON Canada; Department of Medical Oncology, Odette Cancer Centre, Sunnybrook Health Sciences Centre, Toronto, ON Canada; Department of Radiation Oncology, Mayo Clinic, Rochester, MN USA

**Keywords:** Biochemical outcomes, Hypofractionation, Image-guided radiotherapy, Intensity modulated radiotherapy, Prostate cancer, Toxicity

## Abstract

**Background:**

This is the final report of a prospective phase I study which evaluated the feasibility, toxicities, and biochemical control in prostate cancer patients treated with a hypofractionated boost utilizing a fiducial marker-based daily image guidance strategy and small patient-specific PTV margins.

**Methods:**

Low- and intermediate-risk prostate cancer patients underwent transperineal ultrasound-guided implantation of three gold fiducial markers and were treated with three-dimensional conformal radiotherapy to 42 Gy (2 Gy/day). During the first nine fractions of treatment, pre- and post-treatment electronic portal imaging was performed to calculate intrafraction prostate motion. Patient-specific PTV margins were derived and a 30 Gy (3 Gy/day) intensity modulated radiotherapy boost was delivered (Total dose = 72 Gy in 31 fractions; EQD2 = 81 Gy, α/β = 1.4).

**Results:**

Thirty-three patients completed treatment and were followed for a median of 7.2 years (range, 1.2 – 9.5). Seven patients (21%) developed Radiation Therapy Oncology Group (RTOG) late grade 2 GI toxicity and 1 patient (3%) developed late grade 2 GU toxicity. No patients developed late grade 3 GI or GU toxicity. To date, nine patients developed PSA relapse according to the Phoenix criteria. The actuarial five, seven and nine year biochemical control (BC) rates were 87% (95% confidence interval: 69–95), 77% (95% confidence interval: 56–89) and 66% (95% confidence interval: 42–82).

**Conclusions:**

Our study demonstrates that the use of prostate fiducial markers in combination with a daily online image guidance protocol permits reduced, patient-specific PTV margins in a hypofractionated treatment scheme. This treatment planning and delivery strategy was well tolerated in the intermediate time frame. The use of very small PTV margins did not result in excessive failures when compared to other radiation regimens of similar radiobiological intensity.

## Background

Radiotherapy is a widely accepted treatment option for localized prostate cancer. Technological advances in imaging, treatment planning and delivery techniques have transformed radiotherapy. Randomized trials with dose-escalated radiotherapy (74 – 80 Gy) demonstrate an improvement in biochemical control compared to conventional treatment regimens (64 – 70 Gy) [1-9]. However, dose escalation using 3D-CRT is associated with an increase in late rectal toxicity [10].

Investigators have reported a α/β ratio as low as 1.4 for prostate tumours, which is lower than the surrounding rectum and bladder [11-16]. As such, there has been rising interest in using hypofractionated radiotherapy (higher doses per fraction with fewer fractions) to capitalize on the sensitivity of prostate tumours to high fraction sizes while maintaining similar or lowering rates of late normal tissue toxicity. Furthermore, there is the potential for lower costs and improved patient convenience with a shorter course of treatment [17].

Inter- and intrafraction prostate motion is not negligible [18-20]. The essential elements to safely deliver hypofractionated radiotherapy include the utilization of a daily image-guidance strategy and quantifying the geometric uncertainties associated with the treatment technique to derive the optimal planning target volume (PTV) margins to maximize tumour control while limiting late normal tissue toxicity. For this prospective phase I study, we previously presented the feasibility of a fiducial marker-based daily image guidance strategy to deliver a hypofractionated boost to low and intermediate risk prostate cancer patients. We quantified intrafraction prostate motion, derived patient-specific PTV margins and presented the associated acute toxicities of this treatment technique [21]. In this report, we present the late toxicity and efficacy data.

## Methods and materials

### Eligibility criteria

From 2002 to 2003, patients with biopsy proven low- and intermediate-risk adenocarcinoma of the prostate were eligible for this study [22]. These included patients with T1-2, Gleason score ≤ 7, and PSA ≤ 20 ng/mL prostate cancer [23]. Patients with evidence of nodal or distant metastases were ineligible. Written consent was obtained from all patients. This project was approved by the Research Ethics Board of the Sunnybrook Health Sciences Centre.

### Conventional 3D-CRT planning and treatment delivery

Patients underwent transperineal ultrasound-guided implantation of three gold fiducial markers into the prostate, CT simulation in the supine position with a custom vacuum lock bag for immobilization, and digital fluoroscopic imaging of respiratory-induced prostate motion as previously described [21].

The clinical target volume (CTV) consisted of the prostate and the lowest portion of the seminal vesicles directly posterior to the prostate. There was no attempt to include the seminal vesicles above the most superior slice of the prostate. For the first phase of treatment, a 10-mm PTV margin was added to the CTV in all directions to account intra- and interfraction prostate motion. The rectum was contoured as a single solid organ from the bottom of the ischium to the sigmoid flexure. The bladder was contoured as a single solid organ. A conventional four-field 3D-CRT technique was used to deliver a dose of 42 Gy in 21 fractions (2 Gy/day) to the isocenter without daily image guidance. AP and right lateral digitally reconstructed radiographs were developed to document the location of the fiducial markers relative to the treatment field. During the first nine fractions of treatment, pre- and post-treatment electronic portal imaging was performed to calculate intrafraction prostate motion [21].

### Patient-specific PTV margins and intensity modulated radiation (IMRT) boost phase

Using the intrafraction prostate motion data generated from the first phase of treatment, patient-specific PTV margins were generated in the anterior-posterior (AP), superior-inferior (SI) and medial-lateral (ML) directions for the IMRT boost phase [21].

An optimized segmented seven- to nine-field IMRT plan was developed to deliver a dose of 30 Gy in 10 fractions (3 Gy/day) to the patient-specific PTV using inverse planning software (Nomos Corvus). Normal tissue constraints for the IMRT plan were designed to limit the dose to 30% of the rectum and bladder to 60 Gy or less (in equivalent 2-Gy fractions, assuming an α/β value of 2) for the two treatment phases combined. From the two phases combined, the prostate received a total dose of 72 Gy in 31 fractions; an equivalent of 81 Gy in 2 Gy fractions (α/β = 1.4) [13]. These calculations were made using the linear quadratic formula.

Daily image-guidance during the boost phase was achieved by electronic portal imaging in the AP and right lateral direction, and matching the positions of the implanted fiducial markers relative to their initial reference positions on the planning DRR through couch shifts. Only displacements >2 mm were corrected since our own in-house measurements determined the accuracy of the online targeting and correction process to be no less than 2 mm. The IMRT plan incorporated the dose delivered to the prostate and normal tissues from the daily pretreatment imaging.

### Toxicity assessment

Time zero was defined as the start of radiotherapy. Late GI and GU toxicity were assessed using the RTOG/EORTC Late Radiation Morbidity Scheme [24] at 6, 12, 18, and 24 months.

### Biochemical control

For follow-up, patients had a digital rectal examination and serum PSA performed at 3 and 6 months, and then every 6 months for the first 5 years. Thereafter, follow-up was at the discretion of the attending physician. Biochemical failure was defined using the Phoenix definition (nadir + 2 ng/ml) [25].

### Sample size and statistical analysis

At the inception of our study, we estimated the incidence of late grade 2 or greater rectal toxicity between 3% and 14% with our combined 3D-CRT/IMRT treatment technique [26]. Our planned accrual was 30 patients. Time to PSA relapse was defined as the period between the start of radiation treatment and PSA relapse. Patients free from PSA relapse at the end of the study period or by the time they withdrew from the study were censored. The Kaplan-Meier method was used to estimate the PSA relapse-free rate [27]. Statistical analysis was performed in SAS 9.3 (Cary, NC, USA).

## Results

At the time of analysis 33 patients had completed treatment and been followed for a median of 7.2 years (range, 1.2 – 9.5). The majority of patients had intermediate-risk disease (79%) and the remainder had low-risk disease (21%) according to the Canadian consensus on prostate cancer risk stratification (ref). Patient characteristics are summarized in Table 1.

**Table 1:** **Patient characteristics (n = 33)**

**Median age**	**69 years**
	**(Range, 53–80)**
Clinical stage	
T1c	20 (60.6%)
T2a	13 (39.4%)
Gleason score	
6	12 (36.4%)
7	21 (63.6%)
Pretreatment PSA (ng/ml)	
≤10	23 (69.7%)
10 – 20	10 (30.3%)
Risk stratification	
Low	7 (21.2%)
Intermediate	26 (78.8%)

The average PTV margin used during the IMRT hypofractionated boost was 4 mm in the AP direction (range, 2–8 mm), 3 mm in SI direction (range, 2–7 mm), and 3 mm in the RL direction (range, 2–5 mm) [21].

Seven patients (21%) developed late grade 2 GI toxicity and 1 patient (3%) developed late grade 2 GU toxicity. No patients developed late grade 3 GI or GU toxicity after two years of follow-up.

**Figure 1:**
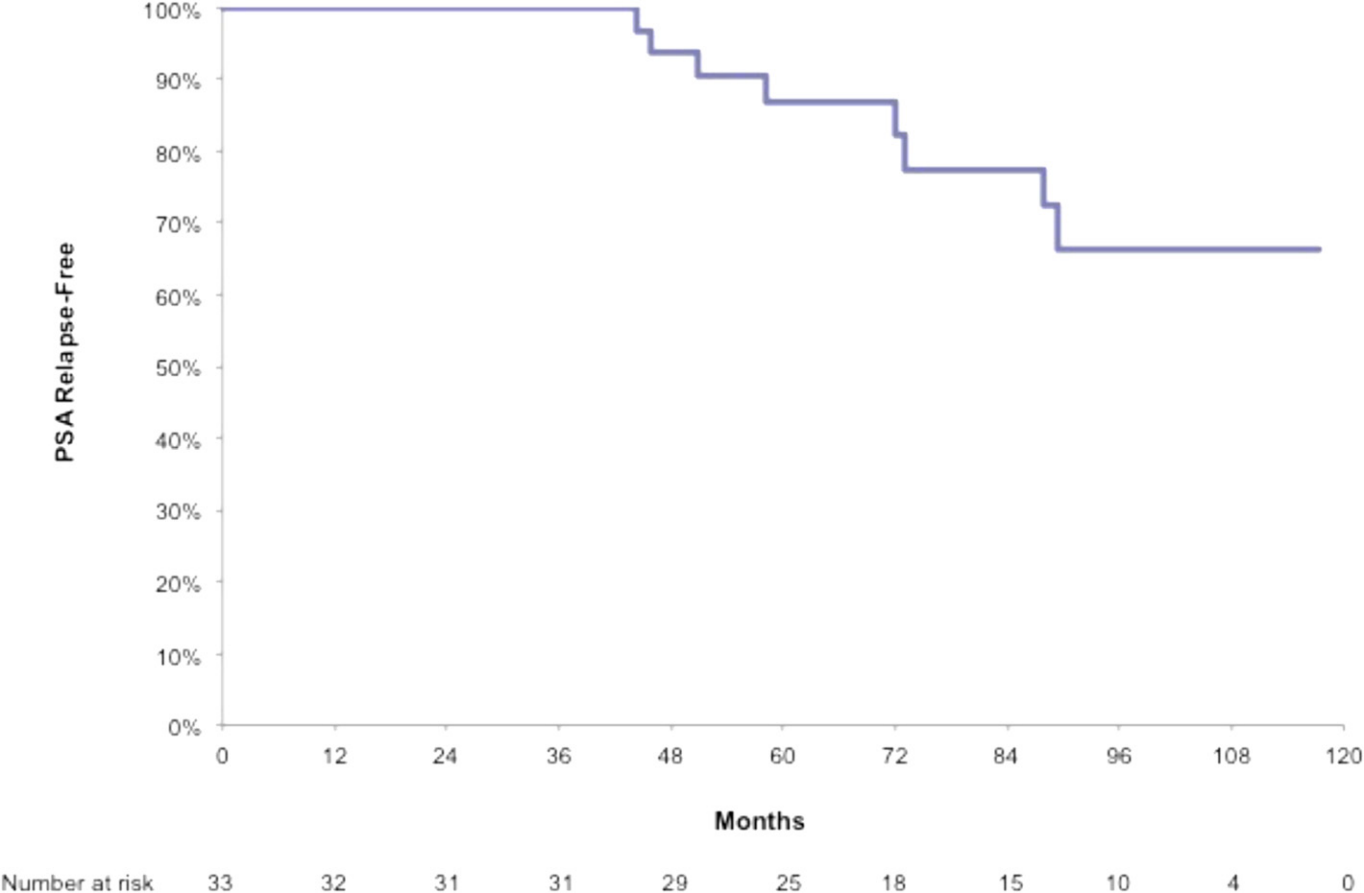
Actuarial analysis of biochemical control.

To date, nine patients developed PSA relapse according to the Phoenix criteria. The actuarial five, seven and nine year biochemical control (BC) rates were 87% (95% confidence interval: 69–95), 77% (95% confidence interval: 56–89) and 66% (95% confidence interval: 42–82) (Figure 1).

## Discussion

We previously showed that the magnitude of intrafraction prostate motion was small (AP 0.72 ± 1.80 mm, SI 0.45 ± 1.27 mm and ML 0.14 ± 0.92 mm), and derived average PTV margins of 4 mm (range 2–8 mm) AP, 3 mm (range 2–7 mm) SI and 3 mm (range 2-5 mm) ML [21]. Since our initial report, additional analyses of prostate fiducial markers with electronic portal images corroborate our findings [28-30]. Kotte et al. [28] showed that margins of at least 2 mm account for intrafraction prostate motion, and Middleton et al. [29] derived PTV margins (AP 3.9 mm, SI 3.2 mm and ML 4.3 mm) that were comparable to ours.

The goal of utilizing a daily-image guidance strategy and reducing the PTV margin is to realize a clinically meaningful difference in treatment toxicity. In the randomized trials of dose escalation the PTV margins used across the studies were heterogeneous, ranging from 5-15 mm. While these studies demonstrated a benefit in biochemical control, there was an increase in late grade 2 or greater GI toxicity (odds ratio of 1.58; 99% CI 1.24–2; p < 0.0001) [31]. In Tables 2 and 3, the PTV margins utilized and the reported late toxicities in the randomized trials of dose escalation [1-9], and contemporary mild hypofractionation [32-36], are summarized alongside the results of the current study for illustrative purposes. Our study was limited by a small sample size and only two years of toxicity follow-up. The variability in the reported toxicities across studies is likely due to differences in the PTV margins, treatment techniques, and dose and fractionation schedules.

**Table 2:** **Summary of randomized trials of dose-escalated external beam radiotherapy**

**Study**	**MDACC (1)**	**Dutch (2, 3,4)**	**PROG (5, 6)**	**MRC RT01 (7, 8)**	**GETUG 06 (9)**
Dose (technique)	78 Gy (4-fld box + 3D-CRT) vs 70 Gy (4-fld box)	78 Gy (4-fld box + 3D-CRT/IMRT) vs 68 Gy (4-fld box)	79.2 GyE (4-fld box + proton boost) vs 70.2 Gy (4-fld box + proton boost)	74 Gy (3D-CRT) vs 64 Gy (3D-CRT)	80 Gy (3D-CRT) vs 70 Gy (3D-CRT)
Median F/U (yrs)	9	5.8	8.9	5.2	5.1
*PTV margins (mm)	Ant/Inf 12.5 -15 Post/Sup 7.5 -10	10 to 68 Gy 5 for 10 Gy boost 0 Post	10 to 50.4 Gy 5 for 28.8 GyE (proton boost)	5 - 10	Ant/Sup/Inf/RL 10 Post 5
*Late toxicity (%)	RTOG	RTOG	RTOG	RTOG	RTOG
≥ Grade 2					
GI	26	35	24	33	20
GU	13	40	29	11	18
Grade 3					
GI	7	5	1	4	6
GU	4	7	2	0	2
Grade 4					
GI	none	1 patient	none	none	none
GU	none	none	none	none	1 patient
Biochemical control (%)	Phoenix	ASTRO	ASTRO	ASTRO	Phoenix
	5 yr - 85 vs 78	7 yr - 54 vs 47	5 yr - 80 vs 61	5 yr - 71 vs 59	5 yr - 76 vs 68
	8 yr - 78 vs 59	Phoenix	10 yr - 83 vs 68		
	10 yr - 73 vs 50	7 yr - 54 vs 45			

MDACC – MD Anderson Cancer Centre; PROG – Proton Radiation Oncology Group; MRC – Medical Research Council; GETUG – Groupe d'Etude des Tumeurs Uro-Génitales.

*High dose arm only.

**Table 3:** **Summary of randomized trials of mild hypofractionated radiotherapy**

**Study**	**MDACC (33)**	**FCCC (34, 35)**	**RENCI (31, 36, 37)**	**UK CHHiP (32)**	**Current study**
Dose/fractions (EQD2 α/β = 1.4)	72 Gy/30 (80Gy) vs 75.6 Gy/ 42 (71 Gy)	70.2 Gy/26 (84 Gy) vs 76 Gy/38	62 Gy/20 (82 Gy) vs 80 Gy/40	60 Gy/20 (77.6 Gy); 57 Gy/19 (73.8 Gy) vs 74 Gy/37	42 Gy/21 plus 30 Gy/10 (81 Gy)
Median F/U (yrs)	4.8	5	2.9	4.2	7.2
*PTV margins (mm)	Not reported	Ant/Sup/Inf/RL7 Post 3	10	PTV1 (80%)	Phase 1 - 10
				Ant/Sup/Inf/RL 10	Phase 2
				Post 10	AP 4
				PTV2 (96%)	SI 3
				Ant/Sup/Inf/RL 10	RL 3
				Post 5	
				PTV3 (100%)	
				Ant/Sup/Inf/RL 5	
				Post 0	
*LateToxicity (%)	RTOG	NR	RTOG	RTOG	RTOG
≥ Grade 2					
GI	11	6	17	3.6 (60 Gy); 1.4 (57 Gy)	21
GU	19	14	14	2.2 (60 Gy); 0 (57 Gy)	3
Grade 3					
GI	3	NR	1 patient	none	none
GU	0	NR	none	none	none
Grade 4					
GI	none	NR	none	none	none
GU	none	NR	none	none	none
Biochemical control (%)	Phoenix	Phoenix	Phoenix		Phoenix
	5 yr - 97 vs 94	5 yr - 86 vs 86	5 yr - 85 vs 79	NR	5 yr - 87
	ASTRO				7 yr – 77
	5 yr - 96 vs 92				9 yr - 66

MDACC – MD Anderson Cancer Centre; FCCC – Fox Chase Cancer Center; RENCI – Regina Elena National Cancer Institute; UK CHHiP - United Kingdom Conventional or Hypofractionated High-dose Intensity Modulated Radiotherapy in Prostate Cancer.

*Hypofractionated arm only; NR – not reported.

Since Brenner and Hall first reported that prostate cancer may have a low α/β ratio [14], there has been growing interest to use hypofractionated treatment regimens to capitalize on the therapeutic ratio that may exist between the sensitivity of prostate cancer to higher doses of radiation per treatment fraction and reduced normal tissue toxicity. The promise of hypofractionation lies in the potential for improved tumour control and increased patient convenience with a shorter overall treatment time and lower costs compared to a standard course of dose-escalated treatment. To date, data from four contemporary randomized trials comparing hypofractionated to dose-escalated radiotherapy do not demonstrate increased biochemical control with higher biologic equivalent doses to the prostate and equivalent late toxicities [34,36]; or the same biochemical control and less late toxicity with isoeffective doses delivered to the prostate as hypothesized [32,33,37-39] (Table 3).

Based on a α/β ratio of 1.4 [13], we delivered an equivalent dose of 81 Gy in 2 Gy fractions. Our pilot study was small, however, it was reassuring to observe that our BC rate was similar to the MD Anderson (8-year Phoenix 78%) [1]; and higher than the UK (5-year ASTRO 71%) [7,8], French (5-year Phoenix 76%) [9] and Dutch (7-year Phoenix 56%) [3,4] dose-escalation trials. In comparison to the hypofractionated treatment arms of the mild hypofractionation trials (Table 3), our BC rate was also similar to the rates reported by Pollack et al. [36] (5-year Phoenix 86%) and Arcangeli et al. [32,37,38] (5-year Phoenix 85%), and lower than the rate reported by Kuban et al. [34] (5-year Phoenix 96%). While these comparisons look favourable, we recognize the wide confidence intervals in our BC rate due to the small sample size. Furthermore, we have not reached a plateau in our biochemical control curve like those seen with brachytherapy where higher biological doses are given [40,41]. This suggests that further biological dose-escalation is warranted. Our group and others have explored more extreme hypofractionation protocols [42-46]. Recent reports of 135 low- and intermediate-risk patients treated with stereotactic body radiotherapy (SBRT; median follow-up 60 months) showed a 5-year bDFS of 99% for low-risk patients and 93% for intermediate-risk patients. Grade 3 or higher toxicities are reported in approximately 1% of patients with 35–40 Gy delivered with 5-fraction SBRT [43,47]. Thus, the benefits of hypofractionation are being realized with doses of 7–8 Gy per fraction.

## Conclusions

Our study demonstrates that the use of prostate fiducial markers in combination with a daily online image guidance protocol permits reduced, patient-specific PTV margins in a hypofractionated treatment scheme. This treatment planning and delivery strategy was well tolerated in the intermediate time frame. The use of very small PTV margins did not result in excessive failures when compared to other radiation regimens of similar radiobiological intensity. We have applied this strategy to our studies of extreme hypofractionation.

## Abbreviations

3D-CRT: Three-dimensional conformal radiotherapy
RTOG: Radiation Therapy Oncology Group
PTV: Planning target volume
CTV: Clinical target volume
IMRT: Intensity modulated radiotherapy
AP: Anterior-posterior
SI: Superior-inferior
ML: Medial-lateral
